# Supplementary material for: Actinomycetes isolated from rhizosphere of wild Coffea arabica L. showed strong biocontrol activities against coffee wilt disease
Source: PLoS One. 2024 Aug 1;19(8):e0306837. doi: 10.1371/journal.pone.0306837 (PMC11293631; doi:10.1371/journal.pone.0306837)
Supplement: S1 Table — (DOCX) [file pone.0306837.s001.docx]

## S1 Table. *In vitro,* inhibition of *Gibberella xylarioides* mycelial growth by rhizobacteria isolates.

| Bacteria isolates | *G. xylarioides* mycelial inhibition (%) | | | Mean | Variance | Std. Deviation | Std. Error of Mean |
| --- | --- | --- | --- | --- | --- | --- | --- |
|  | r1 | r2 | r3 |  |  |  |  |
| MUA13 | 80 | 80.5 | 79.5 | 80.0 | 0.25 | 0.50 | 0.29 |
| MUA14 | 73.8 | 72.2 | 73.6 | 73.2 | 0.76 | 0.87 | 0.50 |
| MUA26 | 82.8 | 83.2 | 83.0 | 83.0 | 0.04 | 0.20 | 0.12 |
| MUA52 | 77 | 76.8 | 76.2 | 76.7 | 0.17 | 0.42 | 0.24 |

r=replicate
