# Supplementary material for: Actinomycetes isolated from rhizosphere of wild Coffea arabica L. showed strong biocontrol activities against coffee wilt disease
Source: PLoS One. 2024 Aug 1;19(8):e0306837. doi: 10.1371/journal.pone.0306837 (PMC11293631; doi:10.1371/journal.pone.0306837)
Supplement: S2 Table — +++: abundant growth, ++: medium growth, +: slow growth, -: No growth. (DOCX) [file pone.0306837.s002.docx]

S2 Table. The growth of selected rhizobacterial isolates under different pH, Temperature, and % of NaCl

| **Isolates** | **pH** | | | | | **NaCl (%)** | | | | | **Temperature(^0^C)** | | | | |
| --- | --- | --- | --- | --- | --- | --- | --- | --- | --- | --- | --- | --- | --- | --- | --- |
|  | 3 | 5 | 7 | 9 | 11 | 0 | 1 | 5 | 7 | 10 | 4 | 25 | 30 | 35 | 40 |
| **MUA_13_** | ++ | +++ | +++ | ++ | - | +++ | ++ | + | + | - | - | ++ | +++ | ++ | + |
| **MUA_14_** | + | + | +++ | + | + | +++ | ++ | + | + | - | - | ++ | +++ | ++ | + |
| **MUA_26_** | + | ++ | +++ | + | + | +++ | ++ | + | + |  | - | ++ | +++ | ++ | + |
| **MUA_52_** | - | ++ | +++ | + | + | +++ | ++ | + | - | - | - | ++ | +++ | ++ | + |

+++: abundant growth, ++: medium growth, +: slow growth, -: No growth
