# Supplementary material for: Actinomycetes isolated from rhizosphere of wild Coffea arabica L. showed strong biocontrol activities against coffee wilt disease
Source: PLoS One. 2024 Aug 1;19(8):e0306837. doi: 10.1371/journal.pone.0306837 (PMC11293631; doi:10.1371/journal.pone.0306837)
Supplement: S3 Table — r = replicate; Control (N): negative control (only sterile distilled water inoculated seedlings); Control (P): positive control (only G. xylarioides infected). (DOCX) [file pone.0306837.s003.docx]

S3 Table. Effect of inoculation of rhizobacteria isolates on reduction of coffee wilt disease incidence caused by *G. xylarioides* under greenhouse conditions.

| Treatment | Disease incidence (DI, %) | | | Mean | Variance | Std. Deviation | Std. Error of Mean |
| --- | --- | --- | --- | --- | --- | --- | --- |
|  | r1 | r2 | r3 |  |  |  |  |
| MUA13+*G. xylarioides* | 27 | 38 | 34.9 | 33.3 | 32.2 | 5.67 | 3.27 |
| MUA14+*G. xylarioides* | 26 | 39.8 | 34 | 33.3 | 48.01 | 6.93 | 4.00 |
| MUA26+*G. xylarioides* | 26 | 38.8 | 35 | 33.3 | 43.21 | 6.57 | 3.80 |
| MUA52+*G. xylarioides* | 60.4 | 74.6 | 65 | 66.7 | 52.49 | 7.25 | 4.18 |
| Control (N) | 0 | 0 | 0 | 0 | 0 | 0 |  |
| Control (P) | 100 | 100 | 100 | 100 | 0 | 0 |  |

r= replicate; Control (N): negative control (only sterile distilled water inoculated seedlings); Control (P): positive control (only *G. xylarioides* infected).
