# Supplementary material for: Actinomycetes isolated from rhizosphere of wild Coffea arabica L. showed strong biocontrol activities against coffee wilt disease
Source: PLoS One. 2024 Aug 1;19(8):e0306837. doi: 10.1371/journal.pone.0306837 (PMC11293631; doi:10.1371/journal.pone.0306837)
Supplement: S4 Table — r = replicate; Control (N): negative control (only sterile distilled water inoculated seedlings); Control (P): positive control (only G. xylarioides infected). (DOCX) [file pone.0306837.s004.docx]

S4 Table. Effect of inoculation of rhizobacteria isolates on reduction of coffee wilt disease severity caused by *G. xylarioides* under greenhouse conditions.

| Treatment | Disease severity (DSI, %) | | | Mean | Variance | Std. Deviation | Std. Error of Mean |
| --- | --- | --- | --- | --- | --- | --- | --- |
|  | r1 | r2 | r3 |  |  |  |  |
| MUA13+*G. xylarioides* | 28.8 | 38.4 | 32.7 | 33.3 | 23.31 | 4.83 | 2.79 |
| MUA14+*G. xylarioides* | 18.5 | 25.5 | 31 | 25 | 39.25 | 6.27 | 3.62 |
| MUA26+*G. xylarioides* | 13 | 16.9 | 20.3 | 16.7 | 13.34 | 3.65 | 2.11 |
| MUA52+*G. xylarioides* | 44 | 50 | 56.1 | 50.03 | 36.60 | 6.05 | 3.49 |
| Control (N) | 0 | 0 | 0 | 0 | 0 | 0 | 0 |
| Control (P) | 100 | 100 | 100 | 100 | 0 | 0 | 0 |

r= replicate; Control (N): negative control (only sterile distilled water inoculated seedlings); Control (P): positive control (only *G. xylarioides* infected).
