# Supplementary material for: Actinomycetes isolated from rhizosphere of wild Coffea arabica L. showed strong biocontrol activities against coffee wilt disease
Source: PLoS One. 2024 Aug 1;19(8):e0306837. doi: 10.1371/journal.pone.0306837 (PMC11293631; doi:10.1371/journal.pone.0306837)
Supplement: S5 Table — r = replicate; Control (N): negative control (only sterile distilled water inoculated seedlings), Control (P): positive control (only G. xylarioides infected). (DOCX) [file pone.0306837.s005.docx]

S5 Table. The biological control efficiency (BE, %) of rhizobacterial isolates against coffee wilt disease caused by *G. xylarioides* under greenhouse conditions.

|  | biological control efficiency (BE, %) | | | Mean | Variance | Std. Deviation | Std. Error of Mean |
| --- | --- | --- | --- | --- | --- | --- | --- |
| Treatment | r1 | r2 | r3 |  |  |  |  |
| MUA13+*G. xylarioides* | 71.2 | 61.6 | 67.3 | 66.7 | 23.31 | 4.83 | 2.79 |
| MUA14+*G. xylarioides* | 81.5 | 74.5 | 69 | 75 | 39.25 | 6.26 | 3.62 |
| MUA26+*G. xylarioides* | 87 | 83.1 | 79.7 | 83.3 | 13.34 | 3.65 | 2.11 |
| MUA52+*G. xylarioides* | 56 | 50 | 43.9 | 49.97 | 36.60 | 6.05 | 3.49 |
| Control (N) | |  |  |  |  |  |  |
| Control (P) | 0 | 0 | 0 | 0 | 0 | 0 |  |

r= replicate; Control (N): negative control (only sterile distilled water inoculated seedlings), Control (P): positive control (only *G. xylarioides* infected).
