# Supplementary material for: Actinomycetes isolated from rhizosphere of wild Coffea arabica L. showed strong biocontrol activities against coffee wilt disease
Source: PLoS One. 2024 Aug 1;19(8):e0306837. doi: 10.1371/journal.pone.0306837 (PMC11293631; doi:10.1371/journal.pone.0306837)
Supplement: Attachment — Submitted filename: CommentsAa.docx [file pone.0306837.s006.docx]

**PONE-D-24-10862**

**General comments:**

1. This study is interesting and demanding for the sustainability of coffee industry.
2. English need to be improved.
3. Found many inconsistencies in words, spelling, formatting etc.
4. Lack of discussion on the findings.
5. No work done on molecular identification or any other appropriate identification on the selected actinomycetes isolates, therefore this manuscript should be rejected.

**Specific comments:**

Line 49, important export?

Line 97, typo Fusarium

Line 107, delete ‘root rhizosphere’, delete root

Line 114, 1376–1890 mas, mas?

Line 116, Mattu is located in the temperate zone

Line 117, ideal for arabica coffee plantation

Line 125-126, describe in detail how field samples can be collected aseptically?

Line 147, fungal agar block, check 1 x 2 cm^2^? Wrong?

Line 147 , what do you mean by leading margin of cultures, please explain.

Line 148, check degree symbol, 25 °C

Line 153, delete ‘free of charge’, was obtained from

Line 162, delete rhizospheric, to actinomycetes isolates with the most.....

Line 181, delete ‘test’ after Catalase, replace with tests after xanthine

Line 183, delete test after methyl red and MacConkey. Typo ‘Methy’

Line 184, delete ‘Cliques’

Line 189, ‘clean? zones’, to ‘clear zones’

Line 199, delete ‘3’ at end line

Line 241, small letter ‘a’ for ‘actinomycetes’ not capital Actinomycetes, and more throughout this manuscript, please check, unless use as starting word in a sentence.

Line 269, typo, ‘seedlings’

Line 290, small letter ‘a’ for actinomycetes, delete ‘root’, rhizospheric soil
